# RETRACTION: Wound incidence and outcomes in atrial fibrillation: Comparing catheter ablation and anti‐arrhythmic drug therapy

**DOI:** 10.1111/iwj.70045

**Published:** 2024-09-03

## Abstract

**Retraction:** XinyuZ. , ShengX. , “Wound incidence and outcomes in atrial fibrillation: Comparing catheter ablation and anti‐arrhythmic drug therapy,” Int Wound J 21, no. 4 (2023): e14612, https://doi.org/10.1111/iwj.14612.38130027 PMC10961873

The above article, published online on 21 December 2023, in Wiley Online Library (http://onlinelibrary.wiley.com/), has been retracted by agreement between the authors; the journal Editor‐in‐Chief, Professor Keith Harding; and John Wiley & Sons, Ltd. The authors submitted a request for retraction, reporting that the conclusions in the published article were partially inaccurate. Following an investigation by the publisher and Editor‐in‐Chief, they concluded that the peer review and publishing process were found to have been manipulated. The retraction has been agreed on because the findings reported in the article are not considered reliable. The authors did not respond to our notice of retraction.

**Retraction:** Z. Xinyu , X. Sheng , “Wound incidence and outcomes in atrial fibrillation: Comparing catheter ablation and anti‐arrhythmic drug therapy,” Int Wound J 21, no. 4 (2023): e14612, https://doi.org/10.1111/iwj.14612.38130027 PMC10961873

The above article, published online on 21 December 2023, in Wiley Online Library (http://onlinelibrary.wiley.com/), has been retracted by agreement between the authors; the journal Editor‐in‐Chief, Professor Keith Harding; and John Wiley & Sons, Ltd. The authors submitted a request for retraction, reporting that the conclusions in the published article were partially inaccurate. Following an investigation by the publisher and Editor‐in‐Chief, they concluded that the peer review and publishing process were found to have been manipulated. The retraction has been agreed on because the findings reported in the article are not considered reliable. The authors did not respond to our notice of retraction.

